# Effect of Comorbid Diabetes on Clinical Characteristics of COVID-19 Patients Infected by the Wild-Type or Delta Variant of SARS-CoV-2

**DOI:** 10.3389/fendo.2022.861443

**Published:** 2022-05-04

**Authors:** Jianguo Zhang, Jinhui Zhang, Zhimin Tao

**Affiliations:** ^1^Department of Emergency Medicine, The Affiliated Hospital, Jiangsu University, Zhenjiang, China; ^2^Department of Critical Care Medicine, The Affiliated Hospital, Jiangsu University, Zhenjiang, China; ^3^Jiangsu Province Key Laboratory of Medical Science and Laboratory Medicine, School of Medicine, Jiangsu University, Zhenjiang, China

**Keywords:** SARS-CoV-2, delta variant, COVID-19, diabetes, vaccination

## Abstract

**Background:**

Diabetes is one of the most common comorbidities in COVID-19 patients that pertains to disease severity, but the causal mechanism regarding its negative impact on COVID-19 outcome has yet been uncovered.

**Methods:**

We retrospectively analyzed 459 COVID-19 patients admitted in early 2020 and 336 COVID-19 patients admitted in August 2021, with their demographic information, medical history, vaccination status (if applied), and laboratory data reported.

**Results:**

Among COVID-19 patients, compared to the non-diabetic group, the diabetic group exhibited elder age, higher proportion of patients with other major comorbidities, more severe dysfunction of innate immune cells, more refractory blood coagulopathy and more detrimental organ damage. For the wild-type SARS-CoV-2 infection, diabetic comorbidity was associated with COVID-19 severity but not mortality, and the glycemic levels in the non-diabetic group upon infection experienced high and analogous to those in the diabetic group. Besides, infected by the delta variant of SARS-CoV-2, the non-diabetic patients did not demonstrate hyperglycemia, and despite different vaccination statuses, the diabetic patients exhibited comparable antibody responses to non-diabetic, showing the robustness of acquired immunity.

**Conclusions:**

SARS-CoV-2 infection may superimpose the deterioration of innate immune systems in diabetic patients, which contributes to their worsened disease outcome, but timely COVID-19 immunization could provide adequate protection in diabetic population that leads to favored prognosis.

## Introduction

Diabetes mellitus (DM) is a common but complicated endocrine disorder, characterized by abnormally high levels of blood sugar (1). Most patients are type 1 or type 2 diabetic, both caused by genetic and environmental factors (2). Type 1 DM (T1DM) is an autoimmune disease in that pancreatic β-cells are self-destructed, so unable to produce insulin, and type 2 DM (T2DM) may own a strong genetic link and can be triggered by various determinants, such as unhealthy lifestyle and high-calorie diet, leading to insulin deficiency or resistance (2). While the incidence of T1DM remains relatively low in China, the prevalence of T2DM has recently been soaring, taking up 12.8% in general population during 2015-2017 (3, 4). In most developed countries, diabetes has been one prevalent non-communicable disease. For instance, in the United States, 10.5% of the population was diabetic in 2018 (5). The rapid growth of diabetic cases across the world has made them an epidemic, levying heavy social and medical burdens (6).

The coronavirus disease 2019 (COVID-19) was announced by the World Health Organization (WHO) a global pandemic on March 11, 2020 (7). It is induced by a novel RNA virus that belongs to the β-coronavirus family, named as severe acute respiratory syndrome coronavirus 2 (SARS-CoV-2) (8, 9). Using angiotensin-converting enzyme 2 (ACE2) as cell entry receptor, SARS-CoV-2 first infects airways and lungs of patients, further resulting in blood coagulopathy and organ disorder (10). This viremia causes more than pulmonary complications and puts those immunocompromised patients at heightened risk (11). Moreover, being a fast-evolving virus, SARS-CoV-2 mutates into several variants, out of which five have been designated as Variants of Concern (VOCs) with much increased transmissibility, including Alpha, Beta, Gamma, Delta, and Omicron variants (12). As of December 26, 2021, the total COVID-19 infection had reached over 278 million with a death rate of 1.9% (12).

Diabetes has been reported as one major comorbidity with SARS-CoV-2 infection. Based on cohort studies of different sizes, the portion of COVID-19 patients with pre-existing diabetes varied from 7.4% to 20% (13–15). Comorbid diabetes was also recognized as a risk factor for worsened COVID-19 progression and outcome (16–19). Nonetheless, the exact mechanism to explain the impact of diabetic comorbidity on the disease course of COVID-19 remains unexplored. Here in this study, we investigate the differences between clinical characteristics of COVID-19 patients with and without diabetes, infected by either wild-type or delta variant SARS-CoV-2, to delineate the role of pre-existing diabetes in COVID-19 disease course.

## Methods

### Patient Information

The study included 459 COVID-19 patients who were hospitalized at the First People’s Hospital of Jiangxia District (FPHJD) in Wuhan City and the Huangshi City Hospital (HCH), both in the Hubei Province, China, during January to April 2020, including 206 patients in the intensive care unit (ICU) and 253 patients in non-ICU isolation ward. In parallel, 336 mild COVID-19 patients infected by the delta variant of SARS-CoV-2 were admitted at the Third People’s Hospital of Yangzhou City (TPHYC), Jiangsu Province, China, in August 2021. COVID-19 patients were confirmed in the study following procedures as previously reported (20, 21). Exclusion criteria were as follows: patients with malignancy, pregnancy, blood disease, or autoimmune disease, and patients who failed to complete blood examinations. The study was approved the Research Ethics Commission of FPHJD, HCH, and TPHYC, respectively. Patient information was anonymous, and written consents were waived. All diabetic patients included in this study were T2DM, defined as a medical history of diabetes or the use of oral hypoglycemic medication or insulin or patients with a fasting glucose ≥7.0 mmol/L or a two-hour postprandial serum glucose ≥11.1 mmol/L, and HbA1c≥6.5%.

### Procedure and Vaccination

COVID-19 patients infected by SARS-CoV-2 or its delta variant were hospitalized and treated as reported (20, 21). Blood cell analysis was conducted by automated hematology analyzer (SYSMEX 800i, Japan; Mindray BC-5300, China), and the biochemical indicator was also analyzed (Toshiba TAB2000, Japan; Beckman AU5800, USA; Roche Cobas 8000, USA). For part of COVID-19 patients admitted in TPHYC in August 2021, two types of inactivated vaccines (Sinovac or Sinopharm) had been administered. A minimal duration of 14 days was considered necessary to develop protective immunity against SARS-CoV-2 infection, so a dose of vaccine was counted effective only if the time between the vaccine shot and the disease onset was longer than 14 days. Serological tests of patients with COVID-19 based on detection of SARS-CoV-2-specific immunoglobulin M (IgM) and immunoglobulin G (IgG) were conducted, using 2019-nCoV IgG chemiluminescence immunoassay (CLIA) microparticles and 2019-nCoV IgM CLIA microparticles (Autobio Diagnostics Corporation Ltd., China).

### Statistical Analysis

All statistical analyses were performed using SPSS version16.0 (SPSS Inc., Chicago, IL). Data were summarized as the median and interquartile range (IQR) for continuous variables values and frequencies for categorical variables. For comparisons between two groups, Mann-Whitney U test was used for continuous variables Categorical variables were examined by χ2 test. Survival curves were plotted using the Kaplan-Meier method and compared between patients with and without diabetes using the log-rank test. All calculated *p* values were two-sided, and *p* values <0.05 were considered statistically significant.

## Results

### Effect of Comorbid Diabetes on Clinical Characteristics of COVID-19 Patients Infected by the Wild-Type SARS-CoV-2

A total of 459 COVID-19 patients infected by the wild-type SARS-CoV-2 were hospitalized in Hubei Province, China, during January-April 2020. Patients were grouped into diabetic or non-diabetic based on the patients’ history of diabetes diagnosis (**Table 1**). Compared to non-diabetic group, diabetic group demonstrated a much higher mean age, but there was no statistical difference between two groups for male predisposition and patient frequency with other major comorbidities that comprise of hypertension, cardiovascular diseases, and bronchitis. Gender susceptibility to COVID-19 has been discussed previously, reaching consensus that the socioeconomic trait of patients (e.g., occupation, smoking history, living styles) may be a determining factor, although sex-specific hormones could also play a role (21). For blood cell counts, the diabetic group possessed significantly higher degree of neutrophilia and anemia (represented by the lower number of RBC and hemoglobin) than non-diabetic group, while the other types of blood cells showed similar levels. For both groups, most coagulation factors and biochemical indictors were comparable despite of diabetic history, except that thrombin time was prolonged and globulin and BUN levels were elevated in the diabetic group, corresponding to more severe viremia including coagulopathy. Of note, the blood glucose levels in both diabetic and non-diabetic groups resembled, revealing that non-diabetic COVID-19 patients experienced episodic hyperglycemia. Reasons for this new-onset hyperglycemia upon COVID-19 hospitalization are many folds, including previously undiagnosed diabetes, usage of non-diabetic medications, and stress-induced transient rise in blood sugar levels (22). Besides, SARS-CoV-2 might attack the ACE2-expressed pancreatic islets to impair the endocrine balance, and the ensuing insulin deficiency/resistance could cause hyperglycemia as other non-COVID-19 critical illness did (23, 24).

**Table 1:** Comparison of clinical characteristics between diabetic and non-diabetic groups infected by SARS-CoV-2 in 2020.

	Diabetic (n=70)	Non-diabetic (n=389)	*p* value
Age, years	62.50 (55.00-73.00)	57.00 (45.00-69.00)	0.010
Male, N (n%)	42 (60.0)	207 (53.2)	0.294
**Comorbidity**			
Hypertension	24 (30.0)	96 (24.7)	0.321
Cardiovascular diseases	10 (14.3)	41 (10.5)	0.359
Bronchitis	2 (2.9)	32 (8.2)	0.114
**Blood cell count**			
WBCs, ×10^9^/L	6.58 (4.95-9.25)	6.40 (4.85-8.50)	0.357
Neutrophils, ×10^9^/L	5.52 (3.94-7.94)	4.60 (3.04-6.79)	0.048
Lymphocytes, ×10^9^/L	0.81 (0.62-1.26)	1.01 (0.65-1.45)	0.111
Monocytes, ×10^9^/L	0.45 (0.26-0.65)	0.45 (0.29-0.62)	0.934
RBCs, ×10^12^/L	3.71 (3.03-4.38)	4.05 (3.50-4.46)	0.035
Hemoglobin, g/L	115 (83-131)	121 (102-136)	0.025
HCT, %	34.4 (27.2-39.3)	36.2 (31.7-40.0)	0.065
Platelets, ×10^9^/L	173 (125-297)	193 (140-260)	0.737
MPV, fL	10.6 (10.1-11.7)	10.7 (10.0-11.5)	0.713
**Coagulation factor**			
Prothrombin time, s	13.3 (12.2-14.2)	13.3 (12.4-14.2)	0.684
INR	1.07 (0.99-1.17)	1.08 (1.01-1.17)	0.839
aPTT, s	30.3 (27.9-33.4)	30.5 (28.2-32.6)	0.849
Thrombin time, s	17.0 (15.7-18.4)	16.3 (15.4-17.4)	0.008
Fibrinogen, g/L	4.04 (3.02-5.20)	3.81 (2.74-4.75)	0.136
D-dimer, mg/L	1.25 (0.32-3.69)	1.05 (0.35-3.17)	0.503
**Biochemical panel**			
CRP, mg/L	28.10 (13.95-65.88)	25.20 (12.55-61.25)	0.373
PCT, ng/mL	1.11 (0.36-1.87)	0.96 (0.36-1.65)	0.379
Total bilirubin, μmol/L	18.20 (10.43-28.58)	17.00 (12.50-27.14)	0.989
Direct bilirubin, μmol/L	8.70 (3.85-14.18)	7.40 (4.20-13.55)	0.851
Indirect bilirubin, μmol/L	10.60 (5.85-17.83)	10.00 (6.65-14.00)	0.454
ALT, U/L	33.25 (20.45-40.45)	32.10 (20.35-42.15)	0.760
AST, U/L	37.05 (22.78-47.93)	31.60 (18.25-45.80)	0.195
ALP, U/L	71.40 (52.75-91.00)	67.00 (52.00-94.60)	0.882
GGT, U/L	42.85 (26.75-63.50)	48.00 (26.30-73.00)	0.642
Total protein, g/L	58.35 (53.83-66.00)	58.10 (52.80-64.35)	0.198
Albumin, g/L	32.50 (28.78-37.73)	33.30 (29.55-37.40)	0.665
Globulin, g/L	26.70 (21.35-30.80)	24.20 (20.00-28.60)	0.022
ADA, U/L	12.70 (10.90-15.90)	14.20 (11.20-18.35)	0.124
BUN, mmol/L	6.60 (4.47-11.73)	5.00 (3.80-8.05)	0.002
Creatinine, mmol/L	72.00 (55.00-82.90)	64.20 (51.80-78.15)	0.050
LDH, U/L	334.50 (205.00-554.25)	358.00 (224.00-516.50)	0.751
Glucose, mmol/L	8.46 (5.48-12.35)	8.73 (6.37-12.74)	0.261
Potassium, mmol/L	4.04 (3.59-4.31)	4.15 (3.57-4.50)	0.275
Sodium, mmol/L	142.30 (137.23-146.50)	142.20 (137.35-147.00)	0.680
Total calcium, mmol/L	1.88 (1.73-1.98)	1.86 (1.62-2.03)	0.739

ADA, adenosine deaminase; ALP, alkaline phosphatase; ALT, alanine aminotransferase; AST, aspartate aminotransferase; BUN, blood urea nitrogen; CRP, C-reactive protein; GGT, γ-glutamyl transferase; HCT, hematocrit; LDH, lactate dehydrogenase; MPV, mean platelet volume; INR, international normalized ratio; PCT, procalcitonin; aPTT, activated partial thromboplastin time; RBC, red blood cell; WBC, white blood cell.

**Figure 1:**
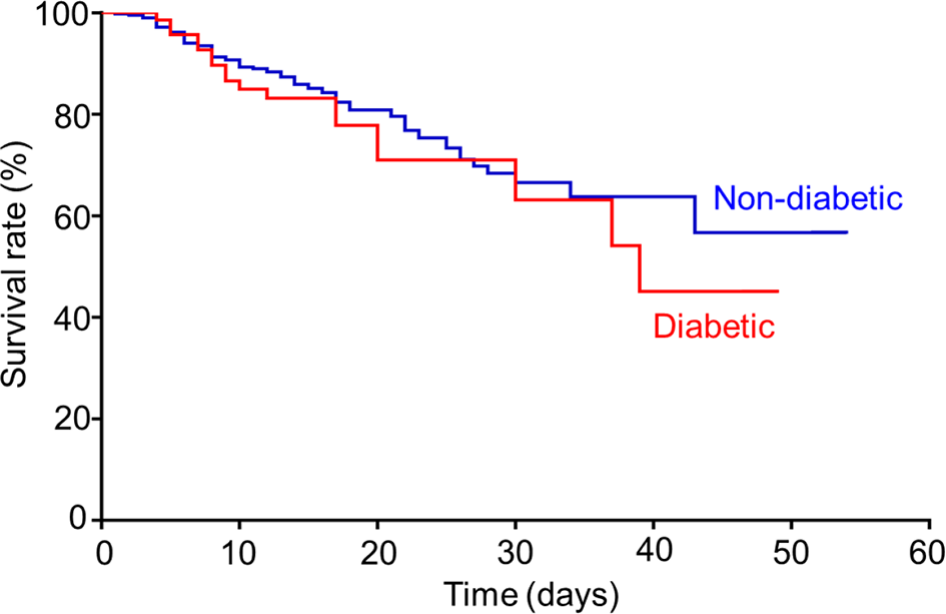
Kaplan-Meier survival curve for COVID-19 patients with or without comorbid diabetes.

For COVID-19 patients infected by the wild-type SARS-CoV-2, 44.9% of total patients were transferred to ICU, where 61.4% of diabetic patients versus (vs.) 41.9% of non-diabetic patients were in severe conditions (*p*=0.002). Consequently, there were mortality rates reported, 25.7% of diabetic patients versus 20.1% of non-diabetic patients (*p*=0.284), respectively. The mean survival duration from hospital admission was 17.6 (3.0-49.0) days in diabetic group and 17.5 (1.0-54.0) days in non-diabetic group, showing no statistical difference. Concurrently, the Kaplan-Meier survival curves (**Figure 1**) depicted a trend toward slightly poorer survival in diabetic group compared to that in non-diabetic group, but no statistical difference was shown between two groups (log-rank test *p*=0.367). Put together, our result here indicated that diabetic comorbidity was closely associated with COVID-19 severity rather than mortality.

### Effect of Comorbid Diabetes on Clinical Characteristics of COVID-19 Patients Infected by the Delta Variant of SARS-CoV-2

A total of 336 COVID-19 patients infected by the delta variant of SARS-CoV-2 were admitted in Yangzhou, Jiangsu Province, China in August 2021. Patients were unvaccinated or immunized with one or two jabs of inactivated COVID-19 vaccines, and their serum antibody productions were examined at time of hospitalization. We then grouped all patients into diabetic or non-diabetic based on their previous diabetes diagnosis (**Table 2**). As a result, compared to non-diabetic group, diabetic group exhibited a much higher age, higher patient frequency with hypertension or cardiovascular diseases, and higher unvaccinated rate. However, antibody responses, though it is indistinguishable to identify whether those antibody productions were due to natural exposure or vaccine immunization, revealed no statistical differences between diabetic and non-diabetic COVID-19 patients, implying the robustness of acquired immunity in diabetic population.

**Table 2:** Comparison of clinical characteristics between diabetic and non-diabetic groups infected by the delta variant of SARS-CoV-2 in 2021.

	Diabetic (n = 31)	Non-diabetic (n = 305)	*p* value
Age, years	63.00 (54.00-72.00)	50.00 (33.00-65.00)	<0.001
Male, N (n%)	17 (54.8)	174 (57.0)	0.813
**Vaccination times**			
0	17 (54.8)	102 (33.4)	0.018
1	5 (16.1)	56 (18.4)	0.759
2	9 (29.0)	147 (48.2)	0.042
**Antibody response**			
None	20 (64.5)	142 (46.6)	0.057
IgG	11 (35.5)	159 (52.1)	0.077
IgM	5 (16.1)	67 (22.0)	0.450
IgG+IgM	5 (16.1)	63 (20.7)	0.550
**Comorbidity**			
Hypertension	22 (71.0)	68 (22.3)	<0.001
Cardiovascular diseases	7 (22.6)	15 (5.0)	0.001
Bronchitis	1 (3.2)	3 (1.0)	0.322
**Blood cell count**			
WBCs, ×10^9^/L	5.22 (4.36-6.84)	5.15 (4.05-6.39)	0.730
Neutrophils, ×10^9^/L	3.03 (2.23-5.04)	3.31 (2.49-4.40)	0.781
Lymphocytes, ×10^9^/L	1.00 (0.70-1.51)	1.08 (0.82-1.47)	0.861
Monocytes, ×10^9^/L	0.52 (0.41-0.67)	0.49 (0.39-0.64)	0.679
RBCs, ×10^12^/L	4.37 (3.94-4.65)	4.50 (4.14-4.92)	0.044
Hemoglobin, g/L	135 (121-139)	137 (124-148)	0.106
HCT, %	38.50 (35.30-41.30)	39.90 (36.80-43.20)	0.068
Platelets, ×10^9^/L	153 (122-178)	172 (134-215)	0.026
MPV, fL	11.0 (10.7-11.6)	11.1 (10.5-11.9)	0.640
**Coagulation factor**			
Prothrombin time, s	11.5 (11.0-12.1)	12.0 (11.5-12.5)	0.002
INR	1.00 (0.95-1.05)	1.05 (1.00-1.09)	0.002
aPTT, s	29.5 (26.8-33.9)	30.2 (27.6-32.8)	0.609
Thrombin time, s	18.0 (17.7-18.8)	17.9 (17.2-18.5)	0.079
Fibrinogen, g/L	3.33 (2.95-3.90)	3.26 (2.74-3.99)	0.509
D-dimer, mg/L	0.41 (0.24-0.53)	0.36 (0.22-0.55)	0.687
**Biochemical panel**			
CRP, mg/L	14.96 (3.31-35.56)	13.52 (4.17-29.06)	0.641
PCT, ng/mL	0.05 (0.04-0.06)	0.04 (0.02-0.05)	0.211
Total bilirubin, μmol/L	8.80 (7.00-14.90)	8.30 (6.00-10.80)	0.109
Direct bilirubin, μmol/L	4.30 (3.10-7.40)	3.80 (2.95-4.90)	0.049
Indirect bilirubin, μmol/L	4.60 (3.10-8.40)	4.30 (3.10-6.15)	0.267
ALT, U/L	24.10 (15.50-40.70)	17.10 (11.50-29.55)	0.014
AST, U/L	26.10 (20.70-41.10)	21.30 (17.35-31.65)	0.018
ALP, U/L	81.00 (67.00-105.00)	80.00 (67.00-96.00)	0.717
GGT, U/L	28.00 (19.00-51.00)	22.00 (14.00-39.00)	0.049
Total protein, g/L	73.90 (69.40-78.10)	72.30 (68.60-76.85)	0.439
Albumin, g/L	46.20 (41.90-48.30)	46.30 (43.70-49.40)	0.331
Globulin, g/L	28.50 (25.30-30.60)	26.50 (23.40-29.00)	0.027
ADA, U/L	16.00 (13.00-18.00)	13.00 (11.00-16.00)	0.002
BUN, mmol/L	5.30 (4.60-7.20)	4.40 (3.40-5.30)	<0.001
Creatinine, mmol/L	78.00 (62.00-108.00)	70.00 (60.00-86.00)	0.056
LDH, U/L	201.00 (185.00-247.00)	197.00 (171.00-236.00)	0.082
Glucose, mmol/L	8.35 (6.48-12.96)	5.75 (4.92-7.29)	<0.001
Potassium, mmol/L	3.61 (3.22-4.16)	3.67 (3.35-3.94)	0.009
Sodium, mmol/L	137.00 (134.00-138.00)	138.00 (136.00-139.00)	0.006
Total calcium, mmol/L	2.30 (2.22-2.36)	2.30 (2.21-2.37)	0.752

In blood test panels, compared to the non-diabetic group, the diabetic group showed the worsened anemia and thrombocytopenia, the elevated levels of ALT, AST, GGT, globulin, ADA and BUN, and the exacerbated hypokalemia and hyponatremia, confirming an intensified viremic effect on COVID-19 patients owing to diabetic comorbidity. Notably, the glucose level in the blood of non-diabetic COVID-19 patients here was significantly decreased, down to a normoglycemic level, suggesting a possibly lessened new-onset impairment in glucose metabolism and endocrine system of COVID-19 patients attacked by the delta variant of SARS-CoV-2. Neither diabetic nor non-diabetic group contained COVID-19 patients further transferred to ICU or deceased.

## Discussion

Compared to normoglycemic patients, hyperglycemic patients with no drug control demonstrated a prolonged length of hospitalization, an elevated chance of ICU transfer, and a heightened rate of in-hospital mortality where infection topped among all causes of death (25, 26). Despite a history of diabetes, intensive insulin therapy to control the blood glucose of hyperglycemic patients in critically ill condition significantly attenuated the inflammation and infection, decreasing the morbidity and mortality (27). In the primary care setting, people with diabetes showed a much higher risk of any infection than those without diabetes (28, 29). Moreover, for community-acquired infections in senior patients (aged ≥ 65) with diabetes, lower respiratory tract infection takes up the highest incidence, while pneumonia and sepsis lead in the fatality causes (30). Particularly, bacterial infection (e.g., *Staphylococcus aureus*, *Mycobacterium tuberculosis*) rather than viral infection was found dominant in the diabetic patients whose increased susceptibility to being infected was ascribed to the impaired immunity at chronic hyperglycemic state (31–33). Out study here confirms that diabetic comorbidity was an independent risk factor for COVID-19 severity.

It has been established that innate host defenses in the diabetic patients are substantially compromised (34). Firstly, the high level of blood glucose can directly glycosylate the protein components in the complement system, so impairing opsonization and suppressing phagocytosis in the early innate immune response (35). Our results echo at this point that diabetic COVID-19 patients demonstrated more abnormality in numbers of blood cells than non-diabetic. Secondly, hyperglycemic condition can induce an array of cellular events, typified by overproduction of advanced glycation end products (AGEs) (36). This further promotes endothelial dysfunction that generates vasoconstriction to alter blood fluidity, and triggers platelet hyperactivity to stimulate thrombus formation, therefore initiating a variety of microvascular and macrovascular complications (e.g., atherosclerosis) (37). This agrees with our finding here that compared to non-diabetic, diabetic patients had higher risks to develop more severe anemia, thrombocytopenia or/and coagulopathy upon COVID-19 infection. Thirdly, through oxidative stress hyperglycemia enhances the circulating concentrations of proinflammatory cytokines, such as tumor necrosis factor-α (TNF-α) and interleukin-6 (IL-6), leading to insulin resistance and resulting in malicious exacerbation of hyperglycemia (38, 39). As a matter of fact, diabetic patients have shown much higher risks of vascular injury than non-diabetic, and ~80% diabetic fatality has been connected to thrombotic events (40). Fourthly, non-alcoholic fatty liver disease (NAFLD), such as non-alcoholic steatohepatitis (NASH), has been found with a dominant prevalence in T2DM patients, where fat accumulation could induce inflammation to cause chronic liver damage (41, 42). This is also evidenced in our results where upon infection of the delta variant SARS-CoV-2, AST, ALT, and GGT values in diabetic patients were significantly higher than those in non-diabetic, indicative of liver damages associated with hyperglycemic T2DM patients. The pre-existing hepatic damages have shown close relevance with worsened prognosis of hospitalized COVID-19 patients (43).

By contrast, the adaptive immunity of diabetic patients has been less characterized. B-cell activation is usually augmented in diabetic patients, followed by a series of T lymphocyte deregulation, such as secretion of pro-inflammatory cytokines, and disoriented T cell differentiation and proliferation, which reflects an impaired profile of diabetes-associated adaptive immunity (44). Having similar metabolic dysfunctions, T1DM patients showed impaired primary antibody response in a T-cell dependent manner while T2DM kept intact responses, suggesting that hyperglycemia might play no role in reduction of antibody production (45). Although glycation of immunoglobin (most through lysine residues in the antigen-binding fragment) has been found frequent in poorly controlled T2DM patients (46), antibody responses in diabetic individuals due to vaccinations against a wide range of infectious diseases (e.g., influenza, hepatitis B) could postpone but yet suffice (47). Likewise, antibody production against SARS-CoV-2 in diabetic patients due to natural exposure or vaccination proves to be still robust, regardless of hyperglycemic state or/and insulin resistance. Different types of antibodies to a variety of SARS-CoV-2 antigens, in terms of antibody level and production time, were shown no substantial difference between diabetic and non-diabetic COVID-19 patients (48). Furthermore, from the hospital admission to 6 months after hospital discharge, the kinetics, endurance, and validation of neutralizing antibody against multiple SARS-CoV-2 antigens were not affected by diabetic comorbidity (49). In parallel, following two doses of mRNA-based COVID-19 vaccines, T2DM individuals demonstrated adequate antibody levels in a comparable manner to those in non-diabetic individuals (50). Our results here stand in line with those findings, as diabetic patients showed comparable antibody responses to non-diabetic in serum after viral infection of COVID-19, independent of vaccination status.

Nevertheless, most studies did not correlate the antibody response to the glycemic control in diabetic individuals. In a recent clinical trial on the immunogenicity of mRNA- or adenovirus vector-based COVID-19 vaccines following two-dose administration in T2DM patients with glycemic monitoring (using HbA1c values), the results indicated that hyperglycemia at time of vaccination worsened the antigen-specific CD4 cell activity and antibody production whereas post-vaccination glycemic control could reverse the situation and improve the immune response (51). The opposite results were also published, demonstrating that upon two-dose COVID-19 vaccination T1DM and T2DM patients had comparable antibody responses to healthy controls, independent of glycemic control (52). Those conflicting reports suggest the exact effect of poor glycemic control on humoral immunity of diabetic patients requires further extensive and in-depth studies to be elucidated. Even so, a good control on glycemic levels for diabetic individuals during COVID-19 vaccination is strongly advised.

Built on the compromised immune system as described above, diabetic comorbidity became a known risk factor for many viral infections (53). Diabetic patients were found with higher prevalence in HIV-positive adults than in general population, where factors were associated with aging subgroup, history of HIV infection, and geometric mean of CD4^+^ T-lymphocyte count (54). Concomitantly, diabetic comorbidity significantly lowered the survival rate of HIV patients with persistent antiviral therapy (55). Immunizations against hepatitis virus infections are recommended for diabetic individuals in the elderly groups, given the fact that the percutaneous injection of insulin intensifies blood exposure to heighten the infection risks and viral hepatitis infections are found with more incidences in diabetic patients, but whether metabolic disorders play a role remains elusive (56). Among respiratory virus infections, diabetes increased the severity and mortality of both pandemic and seasonal influenza patients, but the exact disease outcome may largely differ according to the glycemic variability (57). For coronavirus infection, independent of diabetic history, the blood glucose level was proven to be one prognostic indicator for morbidity and mortality of SARS patients (58). Similarly, the pre-existing diabetes took up the highest prevalence in Middle East respiratory syndrome (MERS) patients, contributing to their severity and fatality (59, 60).

Diabetic vulnerability to the SARS-CoV-2 infection is evident as well. COVID-19 patients of diabetic comorbidity tripled those of non-diabetic in hospital admission, and doubled those of non-diabetes in ICU transfer and in-hospital death (61). Higher glycation of hemoglobin was found to be associated with higher risk of severe disease outcomes, while T1DM than T2DM showed even higher death rate with COVID-19 contraction (19, 62). Reversely, upon COVID-19 infection, good glycemic control in T2DM patients significantly improved their outcome (63–65). As a doorknob for SARS-CoV-2 cell entry, ACE2 is widely expressed in various types of tissues, including pancreatic exocrine and endocrine cells (66). Postmortem examinations in COVID-19 victims spotted SARS-CoV-2 antigens in pancreatic β-cells, displaying that viral binding to ACE2 in human islets impaired the organ function and reduced the insulin secretion (66). On another aspect, ACE2 expressions in total and glycated forms were upregulated in diabetic COVID-19 patients compared to that in non-diabetic peers, which further facilitated the viral entry of SARS-CoV-2 (67). Therefore, this susceptibility to SARS-CoV-2 infection may greatly favor viral invasion and seriously interfere glucose metabolism, aggravating the existing diabetic condition or initiating the new-onset diabetic complication (68, 69). Simultaneously, furin was previously found in a positive proportion to the elevated glucose level in the plasma of diabetic patients, indicative of diabetic risk and mortality (70). As a proprotein convertase, furin cleaves the spike proteins of SARS-CoV-2 and facilitates its host entry (71). Hence, the dysregulated immunity potentiated by comorbid diabetes is a key attribute to the increased infectivity, severity, and mortality in individuals of viral infections including SARS-CoV-2, frequently observed as weakened neutrophil recruitment, reduced lymphocyte activation, and provoked cytokine production, leading to worsened blood coagulopathy and organ damage/failure (21, 24). Notably, insofar no drug has shown significant effects in lowering in-hospital mortality due to COVID-19 infection (72), sending alerts to those at high risks that appropriate vaccination and avoidance of virus exposure (e.g., mask, social distance, etc.) amid the pandemic are of vital importance.

Here our study owns limitations. First, the retrospective and observational design of this study prevents the definition of any cause-effect relationship between the parameters and the outcomes assessed. Second, our study size is small. This further makes the study in diabetic severity and mortality among subgroups of COVID-19 patients even smaller, which renders possible bias. Third, as our research focused on the influence of comorbid diabetes on the clinical manifestation of COVID-19 patients, we were unable to obtain the data of the glycated hemoglobin in all patients and monitor their dynamic changes during the disease courses. This limited our study only to analyze the combined effect of viral infection and diabetic condition upon hospital admission. Similarly, the study is lack of a continuous dataset reporting the changing glucose levels in hospitalized COVID-19 patients in association with the viral clearance process, thereby unable to understand the correlation between viral infection and rise in blood glucose.

## Conclusions

In closing, diabetes being one common comorbidity among COVID-19 patients, we investigated the influence of pre-existing diabetes on the clinical characteristics of COVID-19 patients infected by the wild-type and delta variant SARS-CoV-2 with the underlying machinery. SARS-CoV-2 infection worsens the already compromised host defense in diabetic patients and can cause admission hyperglycemia in non-diabetic patients, further adding to COVID-19 severity. SARS-CoV-2 delta variant could also weaken the diabetic immunity, but immunization due to viral infection or voluntary vaccination imparts comparable antibody responses in diabetic population. For this reason, timely vaccination, avoidance of virus exposure, and good glycemic control, especially to those diabetic seniors, are highly recommended to obviate the infection or prevent the worsened disease outcome once infected.

## Data Availability Statement

The raw data supporting the conclusions of this article will be made available by the authors, without undue reservation.

## Ethics Statement

The studies involving human participants were reviewed and approved by the First People’s Hospital of Jiangxia District (FPHJD), the Huangshi City Hospital (HCH), and the Third People’s Hospital of Yangzhou City (TPHYC). Written informed consent for participation was not required for this study in accordance with the national legislation and the institutional requirements.

## Author Contributions

JGZ and ZT conceived the idea and designed the study. JGZ, JHZ, and ZT contributed to the data processing and table/figure preparation. JHZ and ZT contributed to the statistical analysis. All authors contributed to the manuscript writing and approved the manuscript submission.

## Conflict of Interest

The authors declare that the research was conducted in the absence of any commercial or financial relationships that could be construed as a potential conflict of interest.

## Publisher’s Note

All claims expressed in this article are solely those of the authors and do not necessarily represent those of their affiliated organizations, or those of the publisher, the editors and the reviewers. Any product that may be evaluated in this article, or claim that may be made by its manufacturer, is not guaranteed or endorsed by the publisher.
